# Characteristics of the use of cold combination products among older ambulatory patients at the National Hospital Organization Tochigi Medical Center in Japan: a retrospective single-center observational study

**DOI:** 10.1186/s13104-017-3070-2

**Published:** 2017-12-08

**Authors:** Junpei Komagamine

**Affiliations:** Department of Internal Medicine, National Hospital Organization Tochigi Medical Center, 1-10-37, Nakatomatsuri, Utsunomiya, Tochigi 3208580 Japan

**Keywords:** Cold combination products, Common cold, Elderly, Potentially inappropriate medications

## Abstract

**Objective:**

This study aimed to determine the frequency and characteristics of prescriptions of cold combination products among older ambulatory patients. A retrospective observational study was conducted using electronic medical records. All patients aged 65 years or older who continued visiting internal medicine physicians for at least 1 year were included. The primary outcome was the prescription of cold combination products by any physicians in National Hospital Organization Tochigi Medical Center during a 1-year follow-up.

**Results:**

Seven hundred fifty-six patients were included. The mean age was 75.4 years, 392 (51.9%) were men, the mean Charlson Comorbidity Index was 1.8, and the mean number of medications was 4.9. The proportion of patients who were prescribed cold combination products during the 1-year follow-up was 6.1% (95% confidence interval 4.4–7.8%). The prescription of cold combination products was not significantly associated with age (*p* = 0.11) or Charlson Comorbidity Index (*p* = 0.93) but was associated with an increasing number of medications (*p* < 0.001). A substantial proportion of older ambulatory patients were exposed to cold combination products during a 1-year follow-up.

## Introduction

The common cold is one of the leading reasons for physician visits [1–3]. Many medications for the common cold are available over the counter (OTC) and can be prescribed by physicians [1]. Although several systematic reviews and meta-analyses have reported marginal benefits of several cold medications for symptomatic relief in adults [4–8], they concluded that the balance of benefits and harms needed to be considered when using these medications. Given that non-steroidal anti-inflammatory drugs (NSAIDs) and first-generation H_1_-antihistamines, a type of cold medication, are considered to be potentially inappropriate medications for older patients by several experts [9–11] and that few past studies have included elderly populations [4–7], these two types of medications should be avoided for the common cold in older patients as much as possible due to their potential harms and uncertain benefits.

In Japan, several cold combination products containing caffeine, acetaminophen, NSAIDs and first-generation H_1_-antihistamines are approved for use and are sometimes prescribed by physicians for the common cold [12]. Given the potential harms and uncertain benefits of their use for older patients [4–11], it is important to determine and monitor the frequency of prescription of cold combination products in older patients. Nonetheless, because of the difficulty in obtaining data on patient characteristics and medications [13, 14], the frequency and characteristics of prescriptions of cold combination products have not been studied in Japan. Thus, the aim of this study was to establish the frequency and risk factors associated with the prescription of cold combination products among older ambulatory patients during a 1-year period.

## Main text

### Methods

#### Study design and location

National Hospital Organization (NHO) Tochigi Medical Center is a 350-bed community hospital in the Tochigi prefecture of Japan and one of the two largest acute care hospitals, serving approximately 0.5 million individuals in the area. A retrospective single-center observational study was conducted using the electronic medical records of NHO Tochigi Medical Center from January 2015 to February 2016.

#### Participants and inclusion criteria

All consecutive ambulatory patients aged 65 years or older who had appointments with internal medicine physicians from January 1, 2015 to February 28, 2015 were included. This study included only patients who had three or more visits to internal medicine physicians within a year before the index visit and who had visits to NHO Tochigi Medical Center regularly until at least 1 year after the index visit. Patients with missing data on medications prescribed from other hospitals at the index visit were excluded. During the study period, 963 ambulatory patients at least 65 years old were identified. Two hundred seven patients were excluded for several reasons (96 for a referral to another hospital, 47 for a lack of data on medications prescribed from other hospitals, 38 for loss to follow-up, and 26 for death). Thus, a total of 756 patients were included in the final analysis.

#### Data collection

Data were collected using the electronic medical records of NHO Tochigi Medical Center. Information on age, gender, social insurance, past medical history, Charlson Comorbidity Index (CCI) [15], and medications was retrieved from medical records at the time of the index visit. The total exemption from co-payment was defined as an exemption by social health insurances from co-payment for any medications regardless of diseases. Medications included oral medications, inhalers, and injections, as well as as-needed medications. However, eye drops, intranasal infusers, OTC drugs, and topical medications were excluded, as were medications that were indicated for apparent transient diseases or that were not administered at two or more consecutive visits. Combination drugs that consisted of two or more regulated drugs were counted according to the number of components. For example, a combination drug that consisted of amlodipine and valsartan was counted as two medications.

#### Measurements

The primary outcome was the prescription of cold combination products by any physicians in NHO Tochigi Medical Center during a 1-year follow-up. The prescriptions of cold combination products by physicians in other hospitals were excluded, because this information was not accurate from the database of NHO Tochigi Medical Center. Cold combination products were defined as medical compounds containing all of the following substances: caffeine, acetaminophen, NSAIDs, and first-generation H_1_-antihistamines. The frequency of their prescriptions during the 1-year period was also evaluated. Furthermore, characteristics of physician visits in which cold combination products were prescribed were determined. The documentation of history taking and physical examination for the common cold, the duration of the prescription of cold combination products, other symptomatic medication use, and an antibiotic agent use were evaluated.

#### Statistical analysis

A sample size calculation was not performed prior to data collection. The interval of regular visits of most ambulatory patients to this hospital was within 2 months. Therefore, patients who attended at least one physician visit during 2 consecutive months were screened. The baseline characteristics were described using descriptive statistics. The primary outcome was calculated as the proportion of patients in whom the outcome occurred. The 95% confidence interval (CI) was also calculated for this outcome. For the comparison of baseline characteristics between patients who were prescribed cold combination products and those who were not, Chi squared tests or Fisher’s exact tests were used for categorical variables, and Student’s t-tests were used for continuous variables. To identify the determinants of the prescription of cold combination products, multivariate analysis using binary logistic regression was also conducted to examine the association between selected variables and the primary outcome. The following variables were entered into the logistic regression model: age, gender, total exemption from co-payment, CCI, and number of medications. Total exemption from co-payment was included because past studies have reported that exemption from drug costs was associated with overuse of medications [16, 17]. These analyses were conducted using Excel statistical software package version 2.11 (Bellcurve for Excel; Social Survey Research Information Co., Ltd., Tokyo, Japan), and the level of statistical significance was *p* < 0.05.

## Results

The baseline characteristics of the patients are presented in Table 1. Of the 756 patients, the mean patient age was 75.3 years, 392 (51.9%) were men, the mean CCI was 1.8, and the mean number of total medications was 4.9. The proportion of patients who were prescribed cold combination products during the 1-year follow-up was 6.1% (95% CI 4.4–7.8%). Among the 46 patients prescribed cold combination products, 20 patients (43.5%) were prescribed these products two or more times. Patients who were prescribed cold combination products had a significantly higher number of medications compared with patients who were not (*p* < 0.001). However, there was no difference between the two groups regarding gender, age, total exemption from co-payment, and CCI.

**Table 1:** Characteristics of the ambulatory older patients and comparisons between patients who were prescribed cold combination products and patients who were not

	Total, N = 756	Cold combination products during 1-year follow-up		*p*-value^a^
		Yes, N = 46	No, N = 710	
Age, mean ± SD	75.3 ± 7.3	76.1 ± 7.2	75.3 ± 7.3	0.48
Men, n (%)	392 (51.9)	19 (41.3)	373 (52.5)	0.14
Women, n (%)	364 (48.1)	27 (58.7)	337 (47.5)	0.14
Total exemption from co-payment, n (%)	59 (7.8)	7 (15.2)	52 (7.3)	0.05
Charlson Comorbidity Index, mean ± SD	1.8 ± 1.6	1.8 ± 1.6	1.8 ± 1.6	0.93
Number of medications				
Total, mean ± SD	4.8 ± 2.9	7.0 ± 3.5	4.7 ± 2.7	< 0.001
No medication, n (%)	21 (2.8)	1 (2.2)	20 (2.8)	1.00
1–4 medications, n (%)	362 (47.9)	11 (23.9)	351 (49.4)	< 0.001
5–9 medications, n (%)	319 (42.2)	23 (50.0)	296 (41.7)	0.27
10 or more medications, n (%)	54 (7.1)	11 (23.9)	43 (6.1)	< 0.001
Past medical history, n (%)				
Dementia	41 (5.4)	2 (4.3)	39 (5.5)	0.74
Ischemic stroke	96 (12.7)	5 (10.9)	91 (12.8)	0.82
Myocardial infarction	40 (5.3)	4 (8.7)	36 (5.1)	0.29
Diabetes mellitus	251 (33.2)	12 (26.1)	239 (33.7)	0.29
Chronic kidney disease	248 (32.8)	20 (43.5)	228 (32.1)	0.11
Peptic ulcer	155 (20.5)	5 (10.9)	150 (21.1)	0.09
Frequency of prescription with cold combination products				
Once		26 (56.5)		
Two or three times		10 (21.7)		
Four or more times		10 (21.7)		

^a^Chi squared tests were used for comparisons between patients who were prescribed cold combination products and patients who were not

Table 2 shows the characteristics of 100 visits involving prescriptions of cold combination products among 46 ambulatory older patients during the 1-year follow-up. In most visits, the history and results of the physical examination were not documented. The mean duration of the prescription of cold combination products per visit was 8.8 days. In 42% of all visits, other medications, in addition to these products, were prescribed simultaneously.

**Table 2:** Characteristics of 100 visits with prescription of cold combination products among 46 older ambulatory patients during the 1-year follow-up

Characteristics	Total, N = 100
Specialty of prescribers, n (%)	
Internal medicine	93 (93.0)
Otolaryngology	4 (4.0)
Surgery	3 (3.0)
Medical record documentation^a^, n (%)	
History taking	39 (39.0)
Physical examination	26 (26.0)
Prescription of cold combination products, n (%)	
Duration (days), mean ± SD	8.8 ± 8.5
Simultaneous use of other drugs	
Any use	42 (42.0)
Antibiotics	6 (6.0)
Antitussives	21 (21.0)
Expectorants	9 (9.0)
Exogenous enzymes	7 (7.0)
Acetaminophen	2 (2.0)
Japanese Kampo medicines	14 (14.0)

Cold combination products were defined as medical compounds containing all of the following substances: caffeine, acetaminophen, NSAIDs, and first-generation H_1_-antihistamines

^a^This documentation included information regarding the common cold

Table 3 shows the association between selected variables and prescriptions of cold combination products among older ambulatory patients. In a multivariate analysis using selected variables, only an increasing number of medications was significantly associated with being prescribed cold combination products among older ambulatory patients (adjusted odds ratio (OR) 1.28; 95% CI 1.16–1.41, *p* < 0.001). In post hoc analysis, the ORs of five or more medications and six or more medications for prescriptions of cold combination products were 3.18 (95% CI 1.58–6.40, *p* = 0.001) and 4.57 (95% CI 2.32–9.00, *p* < 0.001), respectively.

**Table 3:** Summary of logistic regression results to predict the prescription of cold combination products

Variables	Odds ratio (95% CI)			
	Univariate	*p*-value	Multivariate^a^	*p*-value
Increasing age	1.01 (0.97–1.06)	0.48	1.00 (0.95–1.04)	0.83
Men	0.64 (0.35–1.16)	0.17	0.59 (0.31–1.12)	0.11
Women	1.57 (0.86–2.88)	0.17	1.68 (0.89–3.18)	0.11
Increasing Charlson Comorbidity Index	1.01 (0.84–1.21)	0.93	0.91 (0.74–1.12)	0.37
Increasing number of medications	1.27 (1.15–1.39)	< 0.001	1.28 (1.16–1.41)	< 0.001
Total exemption from co-payment	2.27 (0.97–5.33)	0.08	1.75 (0.68–4.49)	0.25

^a^The following variables were adjusted: age, gender, CCI, number of medications, and total exemption from co-payment

## Discussion

This study showed that cold combination products were prescribed at least once per year from only a single hospital in 6.1% of older ambulatory patients. Approximately two-fifths of all patients who were prescribed cold combination products were prescribed these products two or more times. Because no data have been published about cold combination products, it was unclear whether these results were worse or better than those of other hospitals in Japan. However, considering that symptomatic therapies for the common cold are prescribed in 2.2% of the general population per year based on a survey in the United States [2] and that older age presents a lower risk of the common cold [18], the annual prescription rate of cold combination products in our hospital seems overly high. Furthermore, in this study, older patients with a higher number of medications were significantly more likely to be prescribed cold combination products, which increased the risk of drug–drug interactions due to polypharmacy [19, 20].

Several possible explanations for these results are considered. First, patients’ expectations increase the medication prescriptions regardless of physicians’ attitudes [16, 21–23]. Moreover, patients often have misconceptions regarding the etiology and treatment of the common cold [24]. These misconceptions might contribute to the excessive expectations of the efficacy of cold medications. Thus, these patients’ expectations might make physicians prescribe cold combination products excessively, although a previous Canadian study reported that the main reason why most patients with cold symptoms seek physicians was not the drug prescription but worries about developing complications [25]. However, it is unclear whether older patients taking more medications have stronger expectations for the prescription. Second, it is possible that a lower cost of medications resulted in the prescriptions. Because of Japan’s medical insurance system, the out-of-pocket cost of OTC cold combination products is higher than that of medications prescribed from hospitals [26]. In fact, this study showed that the total exemption from co-payment, although not significant, tended to increase the prescription of cold combination products. This association is also supported by a past study showing that the exemption from drug charges was an increased risk of prescription among patients consulting general practitioners in the United Kingdom [16]. Third, several prescribers’ factors might affect their prescription of cold combination products, as past systematic reviews reported that physicians’ desire to please patients and their feeling forced to prescribe medications were associated with potentially inappropriate prescribing behaviors [27, 28]. There are also some variations in prescribing habits among physicians [29]. Moreover, physicians’ perceptions of patients’ expectations are sometimes wrong [16]. Patients who physicians perceive as wanting prescriptions are sometimes not hoping for prescriptions. These factors might also contribute to the excessive incidence of prescriptions. However, it is unclear whether physicians prescribing more medications also prescribe cold combination products more frequently. Fourth, older patients with polypharmacy might tend to have a common cold or more severe cold symptoms, although no studies have evaluated the association between polypharmacy and infection [30]. Further studies are needed to clarify these findings.

## Conclusions

A substantial proportion of older ambulatory patients were exposed to cold combination products during a 1-year follow-up. These findings should be confirmed by conducting a multicenter or population-level study in Japan.

## Limitations

First, the study used a retrospective design, which might generate biased data. Moreover, about a fifth of eligible older ambulatory patients were excluded due to loss to follow-up and missing data. Second, the severity of comorbidities was not evaluated. Third, this study was limited to a single center. Therefore, it is also unclear whether these findings were applicable to other hospitals and settings. Fourth, medication adherence was not assessed. Fifth, the true reasons for prescribing cold combination products were not assessed because I had no contact with prescribers. Sixth, this study included only the prescriptions of cold combination products from a single hospital. Seventh, OTC drugs were not assessed. Therefore, the use of cold combination products, including OTC drugs, was likely more frequent. Eighth, the severity of cold symptoms was not assessed. Ninth, the appropriateness of the prescription of cold combination products was not evaluated due to poor medical record documentation by physicians. Moreover, it was unclear how often they were prescribed to patients with cold symptoms. Finally, the association between the prescription of cold combination products and patient outcomes was not evaluated.

## Abbreviations

CCI: Charlson Comorbidity Index
CI: confidence interval
NHO: National Hospital Organization
NSAIDs: non-steroidal anti-inflammatory drugs
OR: odds ratio
OTC: over the counter
SD: standard deviation
